# Non-linear relationship between calf circumference and global cognition in Chinese population: a cross-sectional study of 12,102 Chinese older adults

**DOI:** 10.3389/fnagi.2025.1473135

**Published:** 2025-06-04

**Authors:** Yuting Nie, Cheng Gu, Ruipeng Wu, Fulin Gao, Le Zhang, Yamin Zhang

**Affiliations:** The Department of Neurology, Gansu Provincial Hospital, Lanzhou, China

**Keywords:** calf circumference, global cognition, non-linear relationship, sex difference, CLHLS

## Abstract

**Background:**

Limited evidence exists regarding the relationship between calf circumference (CC) and global cognition in the Chinese population, with minimal research exploring potential sex disparities. Our goal was to investigate the correlation between CC and global cognition using data from the Chinese Longitudinal Healthy Longevity Survey (CLHLS), focusing specifically on sex variations.

**Methods:**

The study participants were older adults who participated in the 2018 CLHLS survey. In this cross-sectional study, we employed multiple linear regression to examine the association between CC and global cognition. Smoothed curve fitting was used to explore the non-linear association between CC and global cognition. Furthermore, subgroup analyses were conducted to evaluate the reliability of the correlation between CC and global cognitive performance.

**Results:**

In total, 12,102 older adults were included in the study. A positive correlation was found between global cognition and CC (β = 0.42, 95% CI = 0.3-0.54, *P* < 0.001) after controlling for confounding factors. Further analysis revealed a non-linear relationship between CC and global cognitive performance. In the overall population, the inflection point for CC was 31 cm; a positive relationship was observed between CC and global cognition for CC values <31 cm (β = 0.177, 95% CI = 0.128-0.225, *P* < 0.001); however, this relationship disappeared for CC values ≥ 31 cm (β: −0.009, 95% CI = −0.04 to 0.023, *P* = 0.591). Furthermore, we identified sex-specific variations in the correlation between global cognitive performance and CC. Notably, among women with CC values <32 cm, a significant positive correlation was observed between CC and overall cognitive function. Conversely, for women with CC ≥ 32 cm, no significant association was found between CC and cognitive performance. Interestingly, no non-linear relationship was detected in males.

**Conclusion:**

This study demonstrated a non-linear relationship between CC and global cognition in older Chinese population. Furthermore, sex disparities are observed in the relationship between CC and global cognition, with a non-linear link evident in women but not in men. Older women with lower CC should actively participate in physical activity to maintain an appropriate CC and prevent cognitive decline.

## 1 Introduction

In China, approximately 6% of individuals aged 60 and above are affected by dementia, and it is estimated that more than 15 million people have dementia (Jia et al., 2020). Dementia imposes substantial economic costs on patients, their families, caregivers and society as a whole, with a national study showing that the annual cost for treating patients with AD in China was $167.74 billion in 2015 and is projected to reach $507.49 billion by 2030 (Aranda et al., 2021; Jia et al., 2018). With the rapidly aging Chinese population, the incidence and economic burden of dementia are expected to increase. Therefore, identifying risk factors for cognitive decline is crucial for slowing the onset and progression of cognitive impairment.

A high prevalence of sarcopenia has been reported in patients with dementia (Chou et al., 2022). Individuals with sarcopenia may be at a higher risk of cognitive decline (Chen et al., 2022; Chang et al., 2016; Beeri et al., 2021). Sarcopenia is closely associated with an increased risk of dementia, and its underlying mechanisms involve multiple processes, including myokines, inflammation, oxidative stress, and insulin resistance. Sarcopenia is often accompanied by a chronic low-grade inflammatory state, and inflammation has been confirmed as a significant risk factor for cognitive decline (Zhao et al., 2024). Individuals with sarcopenia often experience heightened oxidative stress, which can detrimentally impact mitochondrial function, resulting in neuronal loss and consequent cognitive decline (Yu et al., 2024). Furthermore, patients with sarcopenia frequently exhibit insulin resistance, which can impair cognitive function through various mechanisms, including damaging synaptic integrity, increasing Aβ deposition, and promoting tau protein phosphorylation (Kellar and Craft, 2020). Additionally, muscle tissues secrete a variety of myokines that act on the central nervous system to promote neuronal growth and synaptic plasticity, thereby improving cognitive function (Pedersen, 2019; Scisciola et al., 2021). It is noteworthy that physical exercise can enhance cognitive function and decrease the risk of dementia by intervening in the aforementioned pathophysiological processes. Regular exercise has been documented to mitigate chronic inflammation by suppressing the secretion of pro-inflammatory cytokines, boost antioxidant defense mechanisms to mitigate oxidative stress, enhance insulin sensitivity, elevate levels of brain-derived neurotrophic factor (BDNF), irisin, and other muscle factors, thereby enhancing cognitive function (Fernández-Rodríguez et al., 2022; Mahalakshmi et al., 2020; Vints et al., 2022).

The 2019 Asian Sarcopenia Working Group suggests using calf circumference (CC) as a screening method for sarcopenia due to its strong sensitivity and specificity in Asian populations (Chen et al., 2020; Kim S. et al., 2018; Kawakami et al., 2015). CC has been used as a surrogate for assessing skeletal muscle mass (Abdalla et al., 2021; Kiss et al., 2024; Asai et al., 2019). In addition, measuring CC is very simple and non-invasive and is readily available in the community.

Several epidemiological studies have investigated the association between CC and cognitive function, yielding inconclusive results. Some studies have reported a positive correlation between CC and cognitive performance (Won et al., 2017; Kim et al., 2019); indicating that lower CC is linked to a higher risk of cognitive impairment (Liu et al., 2022; Wu et al., 2021; Tai et al., 2021). However, a recent study found no significant correlation between CC and overall cognitive performance (Sampaio et al., 2023). Sex disparities in the relationship between CC and cognitive function have also been documented. For instance, in a study involving Chinese older adults, a stronger association between CC and cognitive decline was observed in female participants (Wu et al., 2021). Conversely, another study reported that reduced CC was only associated with cognitive decline in males, with no such correlation found in females (Kim M. et al., 2018). Sex-specific differences in CC and cognitive function warrant further investigation. Given the inconsistent findings and the limited sample sizes and geographic representation in existing studies, there is a need for comprehensive studies with larger sample sizes encompassing diverse Chinese populations to elucidate the relationship between CC and overall cognitive function. In conclusion, numerous studies in China have examined the relationship between CC and cognitive function in small samples and specific regions, resulting in conflicting conclusions. Therefore, we conducted a cross-sectional study. We hypothesized a non-linear relationship between CC and global cognition, considering sex differences.

## 2 Materials and methods

### 2.1 Study design and participants

Data was collected from the China Longitudinal Healthy Longevity Survey (CLHLS). The CLHLS is a study focused on the health and wellbeing of the older adults in China. It aims to understand their health status and related social, behavioral, and biological factors. The survey encompassed 23 provinces, municipalities, and autonomous regions throughout China, targeting Chinese seniors aged 65 and older and their adult children aged 35-64. The CLHLS, a continuous prospective cohort study, began in 1998 and collects data on the Chinese population through face-to-face interviews by trained staffs, covering approximately 85% of the Chinese population. Approval for the CLHLS study was granted by the Research Ethics Boards at Duke University and Peking University (IRB00001052-13074). The study was carried out by the Declaration of Helsinki.

**FIGURE 1:**
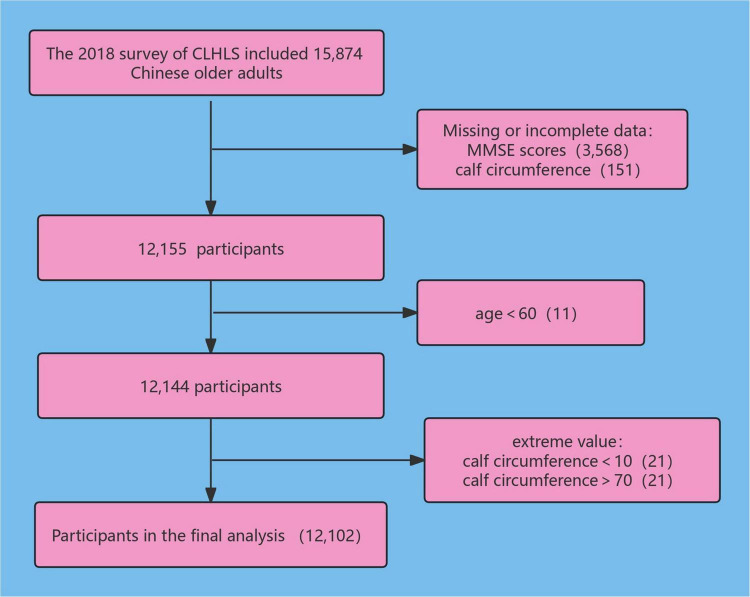
Flowchart of the selection of study population.

We collected CC and cognitive function information from the 2018 waves of the CLHLS. The 2018 survey included 15,874 Chinese older adults, removing 3,568 participants who did not have complete data on MMSE scores (12,306 remaining). Data on 151 cases of missing CC were deleted (12,155 remaining), 11 subjects younger than 60 years old were excluded (12,144 subjects remaining), 21 subjects with CC less than 10 cm were deleted (12,123 remaining), and 21 subjects with CC greater than or equal to 70 cm were deleted, resulting in 12,102 participants being enrolled in this study. The research subject screening process is depicted in Figure 1.

### 2.2 Outcome variable

The CLHLS assesses global cognition using the Brief Mini-Mental State Examination (MMSE), a test with 24 questions. The MMSE includes five aspects of cognitive function (0-30 points): orientation, registration, attention or computation, recall, and language. Its validity and reliability have been proven (Zeng et al., 2002; Zhang et al., 2010). Each question had three possible answers: correct = 1, incorrect = 0, or unanswerable. We coded “unanswerable” responses as incorrect answers based on the previous literature (Li et al., 2017; Yang et al., 2020).

### 2.3 Independent variables

The questionnaire in the 2018 wave of the CLHLS included CC measured in centimeters. Trained investigators measured the CC. Participants sat with their feet placed naturally on the ground. Non-elastic tape was used to measure the CC at the widest part of the calf to avoid compression of the subcutaneous tissue. The detailed CC measurements have been reported in previous studies (Li et al., 2023; Liu et al., 2022).

### 2.4 Covariates

The baseline survey collected socio-demographic data using a structured questionnaire, which included age, weight, height, sex (male/female), education (≤ 9 years/ > 9 years), residence (rural/city), live alone (yes/no), marital status (married and living with spouse/others), smoke (yes/no), drink (yes/no), regular exercise (yes/no), nutritional supplements (yes/no), and common diseases such as hypertension, diabetes, dyslipidemia, stroke. Educational attainment was determined based on self-reported years of schooling. Living alone is measured by the following question: “How many people are living with you?” A response of 0 is defined as living alone. Social events is measured by the question: “Do you take part in some social activities at present?” The responses are coded as follows: (1) almost every day, (2) not daily, but once a week, (3) not weekly, but at least once a month, (4) not monthly, but sometimes, and (5) never. Among these responses, (1) and (2) are defined as “often,” while (3) and (4) are defined as “sometimes.” Marital status is coded as: (1) currently married and living with spouse, (2) separated, (3) divorced, (4) widowed, and (5) never married. In this coding, (1) is defined as “married and living with spouse,” while (3), (4), and (5) are defined as “Other.” The use of nutritional supplements is measured by the question: “Do you usually take nutrient supplements?” Chronic diseases are based on self-reported responses to the question: “Do you suffer from this disease?”

### 2.5 Statistical analysis

R statistical software (version 4.2.2) and Free Statistics analysis platform (Version 1.8, Beijing, China) were utilized for conducting statistical analyses. Demographic characteristics of all participants were categorized based on CC quartiles, with continuous variables presented as mean ± standard deviation or interquartile range. Categorical variables are expressed as percentages. Differences between CC groups were analyzed using one-way ANOVA and chi-square tests. We utilized linear regression models to investigate the effect of CC on global cognition. Model 1 did not include any covariate adjustments. Model 2 adjusts for sex, residence, education, age, marital status and body mass index (BMI). Model 3 accounts for the variables in Model 2 as well as additional factors such as living alone, smoking, drinking, dyslipidemia, hypertension, regular exercise, diabetes, nutritional supplements, heart disease, stroke, cancer, and social events. We used smoothed curve fitting to explore the non-linear association between CC and global cognition. The inflection point was calculated by two step recursive method. We converted CC into categorical variables by quartiles to calculate P for trend. We also conducted further stratified analyses to explore the robustness of the results across subgroups. *P* < 0.05 was deemed statistically significant.

## 3 Results

### 3.1 Clinical characteristics

The study involved 12,102 individuals with an average age of 83.5 ± 11.2 years. Participants were categorized based on the interquartile range of CC, detailed in Table 1 (Q1: 10-27 cm; Q2: 28-30 cm; Q3: 31-34 cm; Q4: 35-68 cm). Individuals in the lowest quartile of CC were more likely to be female (74.5 vs. 38.6%), older (90.3 ± 10.6 vs. 78.6 ± 9.7), have a lower BMI (20.1 ± 4.1 vs. 25.1 ± 4.1), live in rural areas (49.1 vs. 36.7%), be in a state of separation/divorce/widowhood/unmarried (77.4 vs. 41.4%), have less education (94.8 vs. 82.9%), and exhibit poorer cognitive functioning compared to those in the higher CC quartile. They were less likely to be active physically and socially, or take nutritional supplements. Additionally, they had relatively low incidences of hypertension, diabetes, dyslipidemia, heart disease, stroke, tobacco use, and alcohol consumption.

**TABLE 1:** Baseline characteristics of participants.

Calf circumference (cm)	Total	Q1 (10-27)	Q2 (28-30)	Q3 (31-34)	Q4 (35-68)	*p*
Age, mean ± SD	83.5 ± 11.2	90.3 ± 10.6	85.7 ± 10.7	81.2 ± 10.2	78.6 ± 9.7	<0.001***
BMI, Mean ± SD	22.6 ± 4.4	20.1 ± 4.1	21.4 ± 4.0	23.0 ± 3.6	25.1 ± 4.1	<0.001***
Sex, n (%)						<0.001***
Male	5,521 (45.6)	660 (25.5)	1,083 (38.4)	1,755 (51.7)	2,023 (61.4)	
Female	6,581 (54.4)	1,933 (74.5)	1,735 (61.6)	1,640 (48.3)	1,273 (38.6)	
Residence, n (%)						<0.001***
City	6,766 (55.9)	1,319 (50.9)	1,495 (53.1)	1,864 (54.9)	2,088 (63.3)	
Rural	5,336 (44.1)	1,274 (49.1)	1,323 (46.9)	1,531 (45.1)	1,208 (36.7)	
Marital status, n (%)						<0.001***
Married and living with spouse	5,331 (44.1)	587 (22.6)	1,021 (36.2)	1,792 (52.8)	1,931 (58.6)	
Other	6,771 (55.9)	2,006 (77.4)	1,797 (63.8)	1,603 (47.2)	1,365 (41.4)	
Live alone, n (%)						0.568
Yes	423 (3.5)	100 (3.9)	100 (3.5)	108 (3.2)	115 (3.5)	
No	11,679 (96.5)	2,493 (96.1)	2,718 (96.5)	3,287 (96.8)	3,181 (96.5)	
Smoke, n (%)						<0.001***
Yes	1,946 (16.1)	293 (11.3)	435 (15.4)	615 (18.1)	603 (18.3)	
No	10,047 (83.0)	2,279 (87.9)	2,356 (83.6)	2,756 (81.2)	2,656 (80.6)	
NA	109 (0.9)	21 (0.8)	27 (1)	24 (0.7)	37 (1.1)	
Drink, n (%)						<0.001***
Yes	1,841 (15.2)	271 (10.5)	353 (12.5)	572 (16.8)	645 (19.6)	
No	10,086 (83.3)	2,280 (87.9)	2,417 (85.8)	2,785 (82)	2,604 (79)	
NA	175 (1.4)	42 (1.6)	48 (1.7)	38 (1.1)	47 (1.4)	
Education, n (%)						<0.001***
≤ 9 years	10,896 (90.0)	2,457 (94.8)	2,649 (94)	3,056 (90)	2,734 (82.9)	
>9 years	1,206 (10.0)	136 (5.2)	169 (6)	339 (10)	562 (17.1)	
Hypertension, n (%)						<0.001***
Yes	5,401 (44.6)	910 (35.1)	1,132 (40.2)	1,557 (45.9)	1,802 (54.7)	
No	6,701 (55.4)	1,683 (64.9)	1,686 (59.8)	1,838 (54.1)	1,494 (45.3)	
Dyslipidemia, n (%)						<0.001***
Yes	774 (6.4)	74 (2.9)	117 (4.2)	217 (6.4)	366 (11.1)	
No	11,328 (93.6)	2,519 (97.1)	2,701 (95.8)	3,178 (93.6)	2,930 (88.9)	
Diabetes, n (%)						<0.001***
Yes	1,354 (11.2)	190 (7.3)	247 (8.8)	377 (11.1)	540 (16.4)	
No	10,748 (88.8)	2,403 (92.7)	2,571 (91.2)	3,018 (88.9)	2,756 (83.6)	
Cancer, n (%)						0.1
Yes	212 (1.8)	40 (1.5)	41 (1.5)	58 (1.7)	73 (2.2)	
No	11,890 (98.2)	2,553 (98.5)	2,777 (98.5)	3,337 (98.3)	3,223 (97.8)	
Stroke, n (%)						<0.001***
Yes	1,388 (11.5)	210 (8.1)	303 (10.8)	393 (11.6)	482 (14.6)	
No	10,714 (88.5)	2,383 (91.9)	2,515 (89.2)	3,002 (88.4)	2,814 (85.4)	
Heart disease, n (%)						<0.001***
Yes	2,298 (19.0)	397 (15.3)	448 (15.9)	664 (19.6)	789 (23.9)	
No	9,804 (81.0)	2,196 (84.7)	2,370 (84.1)	2,731 (80.4)	2,507 (76.1)	
Nutrient supplements, n (%)						<0.001***
Yes	1,407 (11.6)	244 (9.4)	316 (11.2)	415 (12.2)	432 (13.1)	
No	10,695 (88.4)	2,349 (90.6)	2,502 (88.8)	2,980 (87.8)	2,864 (86.9)	
Social events, n (%)						<0.001***
Often	727 (6.0)	71 (2.7)	110 (3.9)	196 (5.8)	350 (10.6)	
Sometimes	1,103 (9.1)	119 (4.6)	181 (6.4)	311 (9.2)	492 (14.9)	
Never	10,272 (84.9)	2,403 (92.7)	2,527 (89.7)	2,888 (85.1)	2,454 (74.5)	
Regular exercise, n (%)						<0.001***
Yes	4,079 (33.7)	551 (21.2)	828 (29.4)	1,210 (35.6)	1,490 (45.2)	
No	8,023 (66.3)	2,042 (78.8)	1,990 (70.6)	2,185 (64.4)	1,806 (54.8)	
MMSE, Mean ± SD	24.9 ± 6.9	21.3 ± 8.8	24.3 ± 6.9	26.2 ± 5.6	27.1 ± 4.9	<0.001***

BMI, Body Mass Index; MMSE, mini-mental state examination. *p < 0.05,

^**^p < 0.01,

^***^p < 0.001.

### 3.2 Association between calf circumference and cognitive function

**FIGURE 2:**
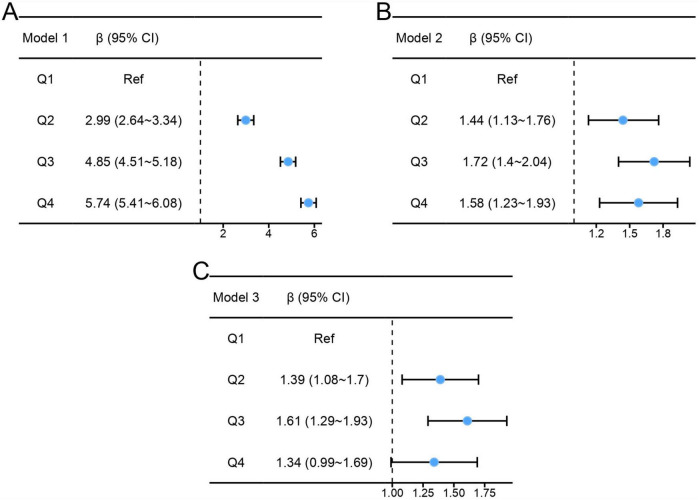
Effect size trend of calf circumference. **(A)** Effect size trend of calf circumference in model 1. **(B)** Effect size trend of calf circumference in model 2. **(C)** Effect size trend of calf circumference in model 3.

**FIGURE 3:**
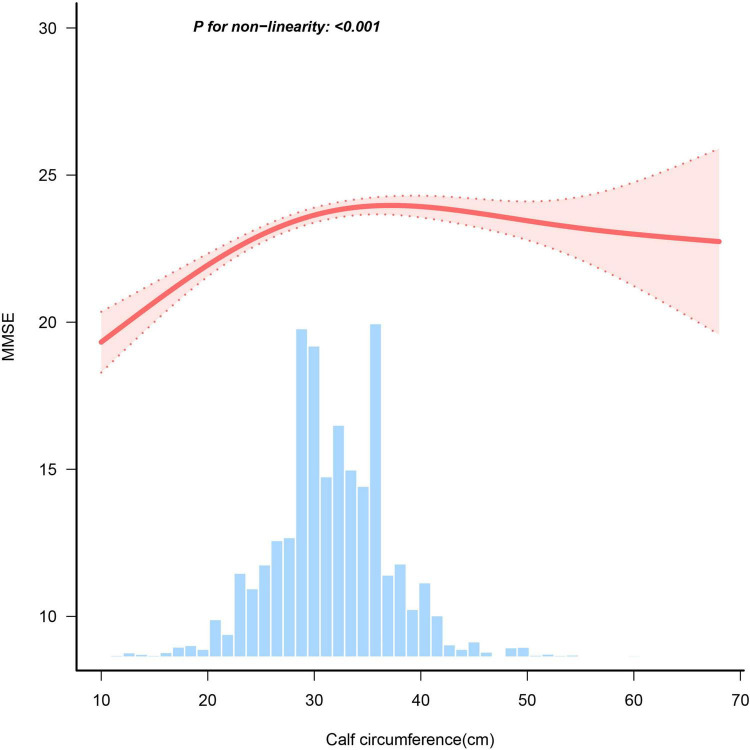
Association between calf circumference and MMSE score. Adjusted for sex, age, residence, education, marital status, BMI, live alone, smoke, drink, dyslipidemia, hypertension, diabetes, heart disease, stroke, cancer, nutrient supplements, regular exercise, social events.

Results of the multiple linear regressions between CC and global cognitive performance are presented in Table 2. CC was positively associated with global cognition in the unadjusted model (β = 1.89, 95% CI: 1.77-2.01, *P* < 0.001). After adjusting for all covariates, the positive association persisted in model 3 (β = 0.42, 95% CI: 0.3-0.54, *P* < 0.001). To investigate a non-linear relationship between CC and global cognition, we converted CC into a categorical variable by quartiles and estimated the trends using sensitivity analyses. The effect size of CC tended to increase before decreasing (Figure 2). In addition, compared with participants in Q1 of CC, the adjusted β in Q2, Q3, and Q4 were 1.39 (95% CI: 1.08-1.7; *P* < 0.001), 1.61 (95% CI: 1.29-1.93; *P* < 0.001) and 1.34 (95% CI: 0.99-1.69; *P* < 0.001). The smoothed curves showed a non-linear relationship between CC and global cognition (Figure 3).

**TABLE 2:** Association between Calf circumference and global cognition.

Variable	Model 1	Model 2	Model 3
	**β (95% CI) *P*-value**	**β (95% CI) *P*-value**	**β (95% CI) *P*-value**
CC (cm) Per SD	1.89 (1.77∼2.01) < 0.001***	0.49 (0.37∼0.61) < 0.001***	0.42 (0.3∼0.54) < 0.001***
**CC quartile**			
Q1 (10-27)	(Ref)	(Ref)	(Ref)
Q2 (28-30)	2.99 (2.64∼3.34) < 0.001***	1.44 (1.13∼1.76) < 0.001***	1.39 (1.08∼1.7) < 0.001***
Q3 (31-34)	4.85 (4.51∼5.18) < 0.001***	1.72 (1.4∼2.04) < 0.001***	1.61 (1.29∼1.93) < 0.001***
Q4 (35-68)	5.74 (5.41∼6.08) < 0.001***	1.58 (1.23∼1.93) < 0.001***	1.34 (0.99∼1.69) < 0.001***
P for trend	1.88 (1.77∼1.99) < 0.001***	0.48 (0.37∼0.6) < 0.001***	0.41 (0.29∼0.52) < 0.001***

CC, Calf circumference; CI, Confidence interval; Ref, Reference. *p < 0.05,

^**^p < 0.01,

^***^p < 0.001.

Model 1: no covariates were adjusted. Model 2: adjust for sex, age, residence, education, marital status, BMI. Model 3: adjust for Model 2 + live alone, smoke, drink, dyslipidemia, hypertension, diabetes, heart disease, stroke, cancer, nutrient supplements, regular exercise, social events.

### 3.3 Threshold effect analysis

We found a threshold of 31 cm for the CC (Table 3). A positive correlation was found between global cognition and CC measurements <31 cm (β = 0.177, 95% CI = 0.128-0.225, *P* < 0.001); however, when CC was ≥ 31 cm, no significant relationship between CC and global cognition was found (β: −0.009, 95% CI = −0.04 to 0.023, *P* = 0.591).

**TABLE 3:** Two-part regression model.

Calf circumference	β	(95% CI)	*P*-value
One-line linear regression model	0.42	(0.3∼0.54)	<0.001***
**Two-piecewise linear regression model**			
Calf circumference<31	0.177	(0.128-0.225)	<0.001***
Calf circumference ≥ 31	−0.009	(−0.04 to 0.023)	0.591
Likelihood Ratio test			<0.001***

Adjusted for sex, age, residence, education, marital status, BMI, live alone, smoke, drink, dyslipidemia, hypertension, diabetes, heart disease, stroke, cancer, nutrient supplements, regular exercise, social events.

*p < 0.05,

^**^p < 0.01,

^***^p < 0.001.

### 3.4 Subgroup analysis

We investigated the stability of the association between CC and global cognitive performance in different subgroups based on sex, age, diabetes, hypertension, heart disease, stroke, residence, and regular exercise (Table 4). Significant interactions were found between sex (interaction *P* < 0.001), age (interaction *P* < 0.001), regular exercise (interaction *P* < 0.001), diabetes (interaction *P* = 0.043), hypertension (interaction *P* = 0.012), and CC. The correlation between CC and global cognition was stronger in females, individuals aged ≥ 85 years, and the older population who did not exercise regularly, and weaker in those with diabetes and hypertension. Other variables including stroke, heart disease, and place of residence did not significantly alter the association between CC and global cognition.

**TABLE 4:** Subgroup analysis of association between calf circumference and global cognition.

Subgroup	Crude.β 95CI%	Crude. P	Adj.β 95CI%	Adj. P	P for interaction
**Sex**					
Male	0.15 (0.12∼0.18)	<0.001	0.02 (−0.01∼0.05)	0.14	<0.001
Female	0.4 (0.37∼0.43)	<0.001	0.11 (0.08∼0.14)	<0.001	
**Age**					
<85	0.07 (0.06∼0.09)	<0.001	0.03 (0.02∼0.05)	<0.001	<0.001
≥ 85	0.33 (0.29∼0.36)	<0.001	0.18 (0.14∼0.22)	<0.001	
**Residence**					
city	0.31 (0.29∼0.34)	<0.001	0.07 (0.05∼0.1)	<0.001	0.167
rural	0.35 (0.32∼0.39)	<0.001	0.07 (0.04∼0.11)	<0.001	
**Regular exercise**					
Yes	0.16 (0.14∼0.19)	<0.001	0.04 (0.01∼0.06)	0.003	<0.001
No	0.36 (0.33∼0.39)	<0.001	0.09 (0.06∼0.11)	<0.001	
**Hypertension**					
Yes	0.26 (0.23∼0.29)	<0.001	0.06 (0.04∼0.09)	<0.001	0.012
No	0.37 (0.34∼0.4)	<0.001	0.08 (0.05∼0.11)	<0.001	
**Diabetes**					
Yes	0.18 (0.13∼0.23)	<0.001	0.05 (0∼0.1)	0.033	0.043
No	0.35 (0.32∼0.37)	<0.001	0.08 (0.05∼0.1)	<0.001	
**Heart disease**					
Yes	0.25 (0.21∼0.3)	<0.001	0.06 (0.02∼0.11)	0.007	0.291
No	0.35 (0.32∼0.37)	<0.001	0.08 (0.05∼0.1)	<0.001	
**Stroke**					
Yes	0.26 (0.2∼0.32)	<0.001	0.04 (−0.02∼0.1)	0.206	0.836
No	0.34 (0.32∼0.36)	<0.001	0.08 (0.06∼0.1)	<0.001	

crude, no covariates were adjusted. Adj: adjust for sex, age, residence, education, marital status, BMI, live alone, smoke, drink, dyslipidemia, hypertension, diabetes, heart disease, stroke, cancer, nutrient supplements, regular exercise, social events.

### 3.5 Sex differences

**FIGURE 4:**
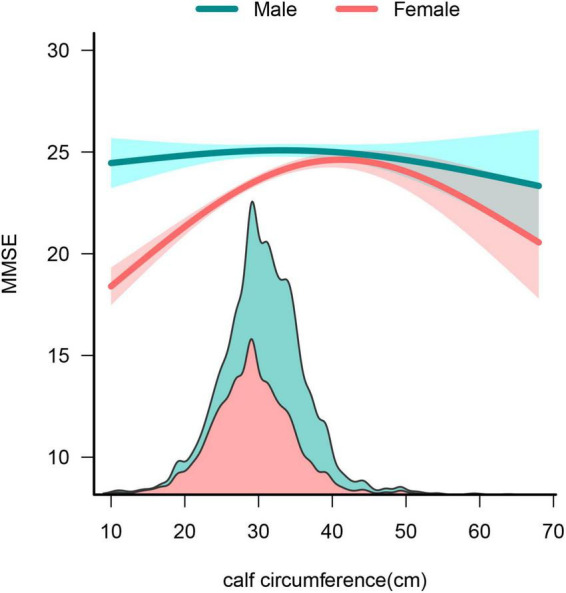
Sex differences in the association between calf circumference and global cognition.

Baseline information stratified by sex is shown in Supplementary Table 1. Table 5 shows the influence of CC on global cognition after adjusting for confounding factors according to sex stratification. Table 6 shows that in males, after adjusting for all covariates, CC was not associated with global cognition in model 3 (β = 0.02, 95% CI: −0.01 to 0.05, *P* = 0.14). Conversely, in females, CC demonstrated a positive correlation with overall cognitive function in Model 3 (β = 0.11, 95% CI: 0.08-0.14, *P* < 0.001). Furthermore, a sex-based stratified curve-fitting analysis was conducted (Figure 4). The stratified curve fitting analysis revealed a non-linear relationship between CC and overall cognitive performance in Chinese older women, while no such relationship was observed in Chinese older men. By employing a two-part regression model, we determined the CC threshold for women to be 32 cm (Table 5). For CC measurements < 32 cm, a significant positive correlation with global cognition was observed in females (β = 0.24, 95% CI = 0.177-0.303, *P* < 0.001); however, when CC was ≥ 32 cm, no significant association between CC and global cognition was detected (β:−0.019, 95% CI = −0.066 to 0.029, *P* = 0.439).

**TABLE 5:** Association between calf circumference and cognitive function stratified by sex.

	Male	Female
	**β (95% CI) *P*-value**	**β (95% CI) *P*-value**
Model 1	0.15 (0.12∼0.18) < 0.001***	0.4 (0.37∼0.43) < 0.001***
Model 2	0.03 (0∼0.06)0.042	0.12 (0.09∼0.15) < 0.001***
Model 3	0.02 (−0.01∼0.05)0.14	0.11 (0.08∼0.14) < 0.001***

Model 1, no covariates were adjusted. Model 2, adjust for age, residence, education, marital status, BMI. Model 3, adjust for Model 2 + live alone, smoke, drink, dyslipidemia, hypertension, diabetes, heart disease, stroke, cancer, nutrient supplements, regular exercise, social events.

*p < 0.05,

**p < 0.01,

***p < 0.001.

**TABLE 6:** Two-part regression model in female.

Calf circumference	β	(95% CI)	*P*-value
One-line linear regression model	0.61	(0.43∼0.79)	<0.001***
**Two-piecewise linear regression model**			
Calf circumference<32	0.24	(0.177-0.303)	<0.001***
Calf circumference ≥ 32	−0.019	(−0.066 to 0.029)	0.439
Likelihood Ratio test			<0.001***

Adjusted for age, residence, education, marital status, BMI, live alone, smoke, drink, dyslipidemia, hypertension, diabetes, heart disease, stroke, cancer, nutrient supplements, regular exercise, social events.

*p < 0.05,

**p < 0.01,

***p < 0.001.

## 4 Discussion

Our study has the largest sample size to investigate the link between CC and overall cognitive abilities in older Chinese population. In the entire population, the CC showed a non-linear relationship with global cognition. CC was positively associated with global cognition when the CC was < 31 cm. Conversely, this relationship disappeared when the CC was > 31 cm. Furthermore, older women exhibited a non-linear relationship between global cognitive function and CC, whereas older men did not show the same correlation.

CC is positively correlated with calf muscle thickness and is a good indicator of muscle mass (Kinoshita et al., 2022; Real et al., 2018; Asai et al., 2019). Our results suggest that CC is positively associated with global cognition when CC is less than 31 cm, whereas it is not related to overall cognitive function when CC is greater than 31 cm. Several studies have reported the relationship between CC and cognitive function. A study of 2,322 older adults in Malaysia found that CC was associated with improved cognitive function (Won et al., 2017). A Japanese study of 1,192 older adults found that individuals with lower CC exhibited poorer cognitive performance, while those with larger CC demonstrated a reduced risk of cognitive decline (Kim et al., 2019). Currently, most studies have found that older adults with lower CC have poorer cognitive function, while higher CC is a protective factor for cognitive impairment (Tai et al., 2021; Ma et al., 2021; Liu et al., 2022; Wu et al., 2021). Most studies agree that CC is positively associated with cognitive function, which is consistent with the findings of our study (when CC is less than 31 cm). In contrast, a study conducted in Brazil involving 263 older adults with an average age of 78 years reported no significant association between CC and cognitive impairment, diverging from our research findings (Sampaio et al., 2023). The variation in the results could stem from the diversity in age and sample size of the study participants. In addition, these studies did not incorporate factors such as nutritional supplements, living alone, or social activities as covariates. In contrast, our study incorporated more covariates and had a larger sample size with a geographically broad study population that was more representative of the Chinese population.

Several factors, including myokines, systemic inflammation, the muscle-gut-brain axis, insulin resistance, and oxidative stress, are implicated in the association between cognitive function and CC (Oudbier et al., 2022; Schlegel et al., 2019). The first mechanism of association between CC and cognitive impairment may be inflammation. Higher systemic markers of inflammation (e.g., CRP, IL-6, TNFα) are associated with lower muscle mass (Tuttle et al., 2020; Can et al., 2017). Persistent inflammation increases the likelihood of developing sarcopenia and dementia (Pan et al., 2021; Xie et al., 2021). In addition, skeletal muscle atrophy may mediate neuroinflammation to affect cognitive function adversely. For instance, muscle atrophy has been shown to impair memory function in young mice, potentially through neuroinflammation induced by muscle-derived hemoglobin (Nagase and Tohda, 2021). Skeletal muscle is capable of secreting various myokines, such as BDNF and irisin, which play crucial roles in the communication between muscle and brain. Recent evidence suggests that myokines produced and released by skeletal muscle can modulate brain function, reducing oxidative stress and neuroinflammation (Scisciola et al., 2021; Rai and Demontis, 2022). Irisin, a myokine released from skeletal muscle, has been shown to enhance BDNF expression, promote synaptic plasticity, increase neuronal survival, and confer anti-inflammatory and antioxidant effects, ultimately enhancing cognition (Peng and Wu, 2022). However, muscle wasting causes postoperative cognitive impairment in rats by reducing BDNF (Nemoto et al., 2022). Moreover, oxidative stress may underlie the association between CC and cognitive function, as evidenced by elevated oxidative stress levels in patients with sarcopenia, which can detrimentally impact neuronal and synaptic function, thereby contributing to cognitive decline (Bellanti et al., 2018). Recent research has highlighted the role of the muscle-gut-brain axis in modulating cognition, potentially linking CC to cognitive function. Muscle factors like irisin and BDNF influence gut microbial composition, promote the growth of beneficial bacteria, support intestinal barrier function, enhance neuroplasticity, mitigate neuroinflammation, and enhance cognitive performance (Cutuli et al., 2023). Additionally, insulin resistance may play a role in the relationship between CC and cognitive function, as it is associated with reduced skeletal muscle mass and represents a risk factor for cognitive decline (Merz and Thurmond, 2020; Ekblad et al., 2017). Peripheral insulin resistance has been implicated in stimulating tau phosphorylation, increasing amyloid-beta toxicity, exacerbating oxidative stress, and compromising synaptic integrity, ultimately contributing to cognitive decline (Kellar and Craft, 2020). It is worth noting that exercise can interfere with pathological factors related to muscle and cognitive function. Physical activity has been shown to decrease inflammatory markers and mitigate age-related oxidative stress (El Assar et al., 2022; Ihalainen et al., 2018). Moreover, regular exercise enhances muscle strength and insulin sensitivity (Grevendonk et al., 2021). Notably, exercise-induced muscle factors facilitate communication between the brain and muscles. For instance, the release of Cathepsin B during exercise can traverse the blood-brain barrier, stimulating BDNF expression in the hippocampus and thereby enhancing spatial memory (Moon et al., 2016). Additionally, exercise influences the gut microbiota and cognitive function through the secretion of muscle factors. Exercise has been proposed to synergistically enhance cognitive function by promoting hippocampal neurogenesis through the upregulation of irisin and BDNF, modulation of inflammation, and promotion of beneficial bacteria and short-chain fatty acids (Schlegel et al., 2019; Cutuli et al., 2023; Morella et al., 2023). Therefore, based on the available evidence, it is advisable for older adults to engage in consistent physical activity and uphold adequate calf circumference to mitigate the likelihood of cognitive decline.

Sex differences in the association between CC and global cognition remain understudied. Our investigation revealed notable sex distinctions in this relationship, particularly in older women. Unlike older men, a non-linear correlation between CC and global cognition was observed in older women. Consistent with our findings, a study by Bing Wu et al. found that the relationship between CC and cognitive impairment was more robust in women (Wu et al., 2021). However, several studies have reported opposite results. A study of older Korean women suggests that CC is not associated with cognitive function (Kim M. et al., 2018). Another large epidemiological study also pointed out that there was no correlation between decreased muscle mass and cognitive decline in older women aged (Abellan van Kan et al., 2013). Our findings do not align with these conclusions. These disparities underscore the need for further well-designed prospective cohort studies across multiple sites to elucidate this issue. The underlying mechanism for these disparities remains unclear. However, the observed variances may stem from fluctuations in sex hormones. Notably, estrogen confers neuroprotective benefits to female nerves by augmenting antioxidant capabilities and synaptic plasticity (Bustamante-Barrientos et al., 2021). Nonetheless, the substantial decrease in estrogen levels post-menopause among women could heighten the susceptibility to cognitive decline (Karamitrou et al., 2023). Moreover, a deficiency in estradiol leads to escalated inflammation (Geraci et al., 2021), which is a contributing factor to cognitive function deterioration. Additionally, there was a general lack of regular exercise in women compared to men in our study (Supplementary Table 2). Movement and exercise are widely acknowledged as protective elements for cognitive function, whereas lack of exercise poses a risk to cognitive health (Northey et al., 2018; Karssemeijer et al., 2017). The lower engagement in physical activity among women could elucidate the sex-based contrast in CC and cognitive function. Furthermore, persistent low-grade inflammation could underlie the sex-specific differences in the association between CC and cognitive function. Women typically manifest more robust immune responses and a heightened susceptibility to autoimmune diseases compared to men (Wilkinson et al., 2022), potentially contributing to a higher incidence of chronic low-grade inflammation in women. Prior research has indicated that increased levels of inflammatory markers influence the correlation between cognitive function and muscle mass in women but not in men (Canon and Crimmins, 2011). Muscle growth inhibitors, proteins produced and released by skeletal muscle, have been identified as promoters of cognitive decline in AD mice (Lin et al., 2019). Previous clinical research revealed a notable increase in myostatin levels in older women compared to older men, suggesting a potential role in sex-related variations in cognitive function and CC (Bergen et al., 2015). Future prospective studies are warranted to confirm this sex disparity, and additional mechanistic investigations are essential to substantiate these findings.

A strength of our study is our study boasts the most extensive sample size among surveys in China focusing on CC and global cognition. Furthermore, in contrast to prior research, our study accounted for a broader range of covariates that had not been fully addressed in previous investigations. These covariates include dyslipidemia, living arrangements, history of cancer, use of nutritional supplements, engagement in social activities, and history of stroke, thereby substantially enhancing the reliability of our findings. Additionally, we conducted curve fitting analyses to assess non-linear associations and performed sensitivity analyses treating CC both as a continuous and categorical variable to bolster the robustness of our results. On the other hand, this work has limitations. First, due to the nature of this cross-sectional study, we were unable to establish a causal link between CC and overall cognitive function. Second, a short epidemiological screening tool (MMSE) was utilized to identify cognitive impairment instead of a comprehensive clinical evaluation. Third, certain variables in our research, like hypertension and diabetes, relied on self-reported information, potentially leading to memory bias. Fourth, other unknown confounders could have affected the results; therefore, more extensive, rigorously designed studies are still needed.

## 5 Conclusion

To the best of our knowledge, this is the first study to discover a non-linear correlation between CC and global cognition. CC showed a strong positive correlation with global cognition below 31 cm, which disappeared above 31 cm. In addition, there were sex-related differences in the non-linear association between CC and global functioning. It is advisable to conduct widespread cognitive screening during routine check-ups for older adults to identify potential cognitive decline and recommend preventative measures early. Additionally, older women with low CC are advised to moderately increase their CC to maintain muscle mass and strength, potentially slowing cognitive impairment through these pathways.

## Data Availability

The datasets presented in this study can be found in online repositories. The names of the repository/repositories and accession number(s) can be found at: https://opendata.pku.edu.cn/dataverse/CHADS?from=timeline&isappinstalled=0.
