# Supplementary material for: Non-linear relationship between calf circumference and global cognition in Chinese population: a cross-sectional study of 12,102 Chinese older adults
Source: Front Aging Neurosci. 2025 Jun 4;17:1473135. doi: 10.3389/fnagi.2025.1473135 (PMC12174059; doi:10.3389/fnagi.2025.1473135)
Supplement: Supplementary file 1 [file Table_1.docx]

Supplementary Material

Supplementary Table 1 Baseline information based on gender stratification

| Variables | Total (n = 12102) | Male  (n = 5521) | Female  (n = 6581) | p |
| --- | --- | --- | --- | --- |
| age, Mean ± SD | 83.5 ± 11.2 | 81.9 ± 10.4 | 84.8 ± 11.6 | < 0.001*** |
| Residence, n (%) | |  |  | 0.103 |
| city | 6766 (55.9) | 3131 (56.7) | 3635 (55.2) |  |
| rural | 5336 (44.1) | 2390 (43.3) | 2946 (44.8) |  |
| Live alone, n (%) | |  |  | 0.002** |
| Yes | 423 ( 3.5) | 162 (2.9) | 261 (4) |  |
| No | 11679 (96.5) | 5359 (97.1) | 6320 (96) |  |
| Regular exercise, n (%) | |  |  | < 0.001*** |
| Yes | 4079 (33.7) | 2160 (39.1) | 1919 (29.2) |  |
| No | 8023 (66.3) | 3361 (60.9) | 4662 (70.8) |  |
| Education, n (%) | |  |  | < 0.001*** |
| ≤9 | 10896 (90.0) | 4714 (85.4) | 6182 (93.9) |  |
| ＞9 | 1206 (10.0) | 807 (14.6) | 399 (6.1) |  |
| Marital status, n (%) | |  |  | < 0.001*** |
| married and living with spouse | 5331 (44.1) | 3301 (59.8) | 2030 (30.8) |  |
| Other | 6771 (55.9) | 2220 (40.2) | 4551 (69.2) |  |
| Drink, n (%) | |  |  | < 0.001*** |
| Yes | 1841 (15.2) | 1462 (26.5) | 379 (5.8) |  |
| No | 10086 (83.3) | 4002 (72.5) | 6084 (92.4) |  |
| NA | 175 ( 1.4) | 57 (1) | 118 (1.8) |  |
| Smoke, n (%) | |  |  | < 0.001*** |
| Yes | 1946 (16.1) | 1671 (30.3) | 275 (4.2) |  |
| No | 10047 (83.0) | 3812 (69) | 6235 (94.7) |  |
| NA | 109 ( 0.9) | 38 (0.7) | 71 (1.1) |  |
| BMI, Mean ± SD | 22.6 ±4.4 | 22.8 ± 4.1 | 22.4 ±4.6 | < 0.001*** |
| Hypertension, n (%) | |  |  | < 0.001*** |
| Yes | 5401 (44.6) | 2332 (42.2) | 3069 (46.6) |  |
| No | 6701 (55.4) | 3189 (57.8) | 3512 (53.4) |  |
| Diabetes, n (%) | |  |  | 0.018* |
| Yes | 1354 (11.2) | 577 (10.5) | 777 (11.8) |  |
| No | 10748 (88.8) | 4944 (89.5) | 5804 (88.2) |  |
| Cancer, n (%) | |  |  | 0.462 |
| Yes | 212 ( 1.8) | 102 (1.8) | 110 (1.7) |  |
| No | 11890 (98.2) | 5419 (98.2) | 6471 (98.3) |  |
| Social events, n (%) | |  |  | < 0.001*** |
| often | 727 ( 6.0) | 368 (6.7) | 359 (5.5) |  |
| sometimes | 1103 ( 9.1) | 637 (11.5) | 466 (7.1) |  |
| never | 10272 (84.9) | 4516 (81.8) | 5756 (87.5) |  |
| Nutrient supplements, n (%) | |  |  | < 0.001*** |
| Yes | 1407 (11.6) | 575 (10.4) | 832 (12.6) |  |
| No | 10695 (88.4) | 4946 (89.6) | 5749 (87.4) |  |
| Heart disease, n (%) | |  |  | < 0.001*** |
| Yes | 2298 (19.0) | 957 (17.3) | 1341 (20.4) |  |
| No | 9804 (81.0) | 4564 (82.7) | 5240 (79.6) |  |
| Dyslipidemia, n (%) | |  |  | 0.025* |
| Yes | 774 ( 6.4) | 323 (5.9) | 451 (6.9) |  |
| No | 11328 (93.6) | 5198 (94.1) | 6130 (93.1) |  |
| Stroke, n (%) | |  |  | 0.006** |
| Yes | 1388 (11.5) | 681 (12.3) | 707 (10.7) |  |
| No | 10714 (88.5) | 4840 (87.7) | 5874 (89.3) |  |

*p < 0.05， **p < 0.01， ***p < 0.001
